# Validation of the modified Skåne emergency department assessment of patient load (mSEAL) model for emergency department crowding and comparison with international models; an observational study

**DOI:** 10.1186/s12873-021-00414-6

**Published:** 2021-02-22

**Authors:** Jens Wretborn, Håkan Starkenberg, Thoralph Ruge, Daniel B. Wilhelms, Ulf Ekelund

**Affiliations:** 1 Department of Emergency Medicine, Local Health Care Services in Central Östergötland, Linköping, Östergötland Sweden; 2 Department of Clinical Sciences Lund, Emergency Medicine, Faculty of Medicine, Lund University, Lund, Sweden; 3 Enköping Hospital, Region Uppsala, Sweden; 4 Department of Emergency Medicine Solna, Karolinska Institutet, Solna, Sweden; 5 Department of Emergency and Internal Medicine, Skåne University Hospital, Malmö, Sweden; 6 Department of Clinical Sciences Malmö, Faculty of Medicine, Lund University, Lund, Sweden; 7 Department of Biomedical and Clinical Sciences, Faculty of Health Sciences, Linköping University, Linköping, Sweden

**Keywords:** Emergency department, Crowding, Boarding

## Abstract

**Background:**

Emergency Department crowding is associated with increased morbidity and mortality but no measure of crowding has been validated in Sweden. We have previously derived and internally validated the Skåne Emergency Department Assessment of Patient Load (SEAL) score as a measure of crowding in Emergency Departments (ED) in a large regional healthcare system in Sweden. Due to differences in electronic health records (EHRs) between health care systems in Sweden, all variables in the original SEAL-score could not be measured reliably nationally. We aimed to derive and validate a modified SEAL (mSEAL) model and to compare it with established international measures of crowding.

**Methods:**

This was an observational cross sectional study at four EDs in Sweden. All clinical staff assessed their workload (1–6 where 6 is the highest workload) at 5 timepoints each day. We used linear regression with stepwise backward elimination on the original SEAL dataset to derive and internally validate the mSEAL score against staff workload assessments. We externally validated the mSEAL at four hospitals and compared it with the National Emergency Department Overcrowding Score (NEDOCS), the simplified International Crowding Measure in Emergency Department (sICMED), and Occupancy Rate. Area under the receiver operating curve (AuROC) and coefficient of determination was used to compare crowding models. Crowding was defined as an average workload of 4.5 or higher.

**Results:**

The mSEAL score contains the variables *Patient Hours* and *Time to physician* and showed strong correlation with crowding in the derivation (r^2^ = 0.47), internal validation (r^2^ = 0.64 and 0.69) and in the external validation (r^2^ = 0.48 to 0.60). AuROC scores for crowding in the external validation were 0.91, 0.90, 0.97 and 0.80 for mSEAL, Occupancy Rate, NEDOCS and sICMED respectively.

**Conclusions:**

The mSEAL model can measure crowding based on workload in Swedish EDs with good discriminatory capacity and has the potential to systematically evaluate crowding and help policymakers and researchers target its causes and effects. In Swedish EDs, Occupancy Rate and NEDOCS are good alternatives to measure crowding based on workload.

## Introduction

Emergency Department (ED) crowding is a prevalent problem in many health care systems and associated with increased morbidity, mortality and decreased quality of care [1, 2]. There are several measures of crowding, from simple numeric scores to more complex models which incorporate different measures into a single score [3]. The rationale is that complex models encompass different aspects of crowding and are thought to provide better information compared to single variables such as *Occupancy Rate* [4, 5]. Crowding in Swedish EDs has previously been limited [6], but has become a problem in recent years, and several research projects on the topic have been initiated [7, 8]. However there is no definition or validated measure of crowding in Sweden which limits the efforts to detect and systematically prevent its negative effects on patient care. Internationally the National Emergency Department Overcrowding Score (NEDOCS) have been derived and validated in the US using staff perception of crowding and generates a score from 1 to 200, with overcrowding defined as a score over 100 [5]. The International Crowding Measure in Emergency Department (ICMED) was derived in the UK against staff perception of crowding and danger. Points are assigned to different aspects of ED care on a scale from 1 to 7, and a score of 3 or higher has a high predictive ability for crowding [4]. Both scores include variables that are not readily available in the ED and there are concerns whether they could be applied to the Swedish healthcare system [9].

Since there are no validated measures of crowding in Sweden, we derived and validated the Skåne Emergency Department Assessment of Patient Load (SEAL) score in a large regional health care system in southern Sweden [9]. We used staff perception of workload as a measure of ED crowding since it is reasonable to assume that a substantial part of the negative effects of crowding are due to high staff workload, and since similar measures have been shown to be reliable in other crowding models like NEDOCS and ICMED [4, 5]. The score is intended for automated calculation based on data from the EHR and internal validation has shown consistent results [9]. So far, the SEAL score has not been externally validated, and its utility has not been compared with existing international crowding models.

Sweden, with a population of 10 million, has a universal publicly funded health care system granting emergency care with a small co-payment at 72 EDs spanning from small rural EDs to large urban academic EDs. The health care is organized into 21 health care systems with different EHRs and ED support systems. Because of differences in the EHRs, all variables in the original SEAL score cannot be measured reliably in many Swedish health care systems at this time. For optimal usability and generalisability, a crowding model needs to include measures readily available in the EHR at many EDs.

### Aim

In the present study, we aimed to derive and externally validate a modified SEAL model (mSEAL) to measure ED crowding based on variables reliably available across different EHRs, and to compare it with established international measures of crowding.

## Methods

### Derivation and validation of mSEAL

We used linear regression analysis with stepwise backward elimination on the original SEAL-dataset [9]. The SEAL model includes the variables *Patient Hours, High acuity Patients, Time waiting for a physician* and *Occupancy rate.* In short, *Patient hours* is the sum of time all patients spent in the ED during the past hour, measured in hours, and has the largest impact on the model score. *High Acuity Patients* is the proportion of high (highest and second highest out of five categories) acuity patients at the ED. *Time waiting for a physician* is the time from triage and nursing procedures to first physician contact. *Occupancy rate* is the number of ED patients divided by the number of ED treatment beds.

Since *High Acuity Patients* and *Time waiting for a physician* could not be reliably collected at all EDs, these were left out and exchanged for the new variable *time to physician* (measuring the time from registration in the ED to first physician contact) to form the mSEAL score. The mSEAL was then internally validated in a separate data collection from two of the five hospitals in the original study, as well as externally validated at four EDs, as described below.

### External validation

We collected data at four EDs in Sweden (Table 1) during September to November 2017; Three EDs at academic teaching hospitals and one at a rural community hospital in two different healthcare systems. Workload assessments, rated on a Likert scale from 1 (no workload) to 6 (very high workload), were collected from each included ED at 5 pre-defined time points per day (08:00, 12:00, 16:00, 20:00, 23:00). These time points were selected to represent known periods of both high and low levels of crowding, and were separated in time to reduce the risk of temporal correlation between assessment and scores [10, 11]. All working staff (doctors, nurses and enrolled nurses) rated their workload during the previous hour on a questionnaire.

**Table 1:** Characteristics of included study sites

Hospital	Linköping University Hospital	Motala Community Hospital	Karolinska University Hospital at Solna	Karolinska University Hospital at Huddinge
**Hospital beds**	550	100	700	800
**Trauma level**	1	3	1	1
**Hospital type**	Academic teaching hospital	Rural Community	Academic teaching hospital	Academic teaching hospital
**ED beds**	48	16	43	48
**Annual ED visits**	50,000	25,000	77,000	75,000

All ED patient flow data were collected after the assessment period from the electronic health records (EHR) at each hospital; TakeCare™ at Karolinska University Hospital Solna and Huddinge, and Cambio Cosmic™ at Linköping University Hospital and Motala Community Hospital. Workload assessments were transcribed from paper questionnaires to digital spreadsheets.

### International models

To compare the mSEAL model with established measures of crowding we computed the Occupancy rate [12] for each time point at all EDs. We also calculated the simplified ICMED (sICMED) [13] and NEDOCS [5], with some minor modifications (see below) for each time point at the two EDs (Linköping and Motala) where the needed data were available. The variable occupied ventilator/trauma bay was excluded from the present NEDOCS calculation since the data could not be reliably calculated from the EHRs. We used the short version of ICMED (sICMED) score since the variable Left without being seen was not registered systematically in the EHRs at the study sites [13]. We also excluded ambulance offload time since this does not occur in any of the study sites and very rarely in any Swedish ED. Based on the result of our SEAL derivation study [9], and recent evidence suggesting that negative effects on patient care occur mostly at high crowding levels [14], a mSEAL score in the top quartile (over 4.5) was defined as crowding.

### Ethics

The study was approved by the regional ethics committee at the county of Östergötland and Stockholm.

### Statistical analysis

Linear regression was used to assess correlation and stepwise backward elimination was used to derive the mSEAL model. Correlations were reported as the coefficient of determination (r^2^) with general qualitative descriptions [15]. Area under the receiver operating curve (AuROC) with Youden Index [16] was used to describe the models test characteristics. A two-tailed *p*-value of less than 0.05 was considered statistically significant. Data was imported into Pandas (v 0.23) [17] and analyzed with Python using the Scipy library (v 1.17) [18] and Statsmodels library (v. 0.10) [19].

## Results

### Derivation and internal validation

The mSEAL included the variables *Time to MD* and Patient Hours; mSEAL = 1.49 + 9.72 * Patient Hours + 0.18 * Time to MD with a range from 1 to 6. The mSEAL model showed similar correlation with the assessments (r^2^ = 0.47) as the original SEAL model in the derivation dataset (r^2^ 0.47 vs 0.51) and the internal validation dataset (r^2^ 0.69 and 0.66 vs 0.64 and 0.64).

### External validation

**Fig. 1:**
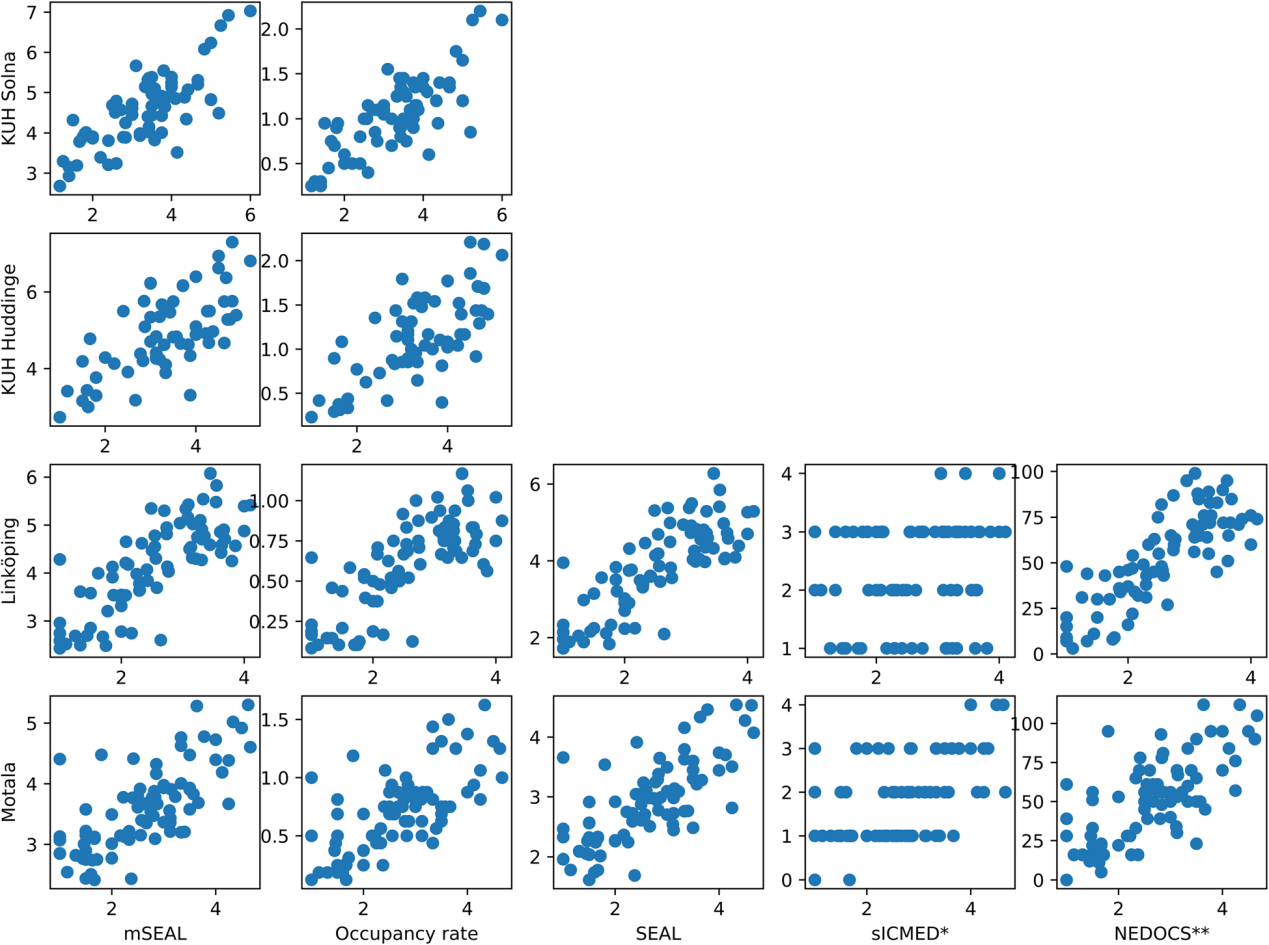
Correlations between assessed workload and crowding score at each study site. mSEAL, modified Skåne Emergency Department Assessment of Patient Load. SEAL, Skåne Emergency Department Assessment of Patient Load. sICMED, short International Crowding Measure in Emergency Departments NEDOCS, National Emergency Department Overcrowding Score. * Ambulances waiting to offload patients not calculated. ** Respirator/Trauma room variable not calculated

A total of 2794 Workload assessments were collected at the four study sites on 333 (95%, 17 missing) timepoints. As can be seen in Table 2, the mSEAL score and Occupancy rate was possible to calculate at all EDs, and both showed a strong correlation to staff workload. NEDOCS compared well with both Occupancy rate and mSEAL scores, while sICMED performed worse at both hospitals. Scatterplots of scores against workload assessments were similar for all models and EDs except for sICMED that uses discrete values (Fig. 1).

**Table 2:** Correlations (r^2^) between assessed workload and crowding score at each ED

Emergency Department	mSEAL	Occupancy Rate	sICMED*	NEDOCS**
Solna	0.60	0.49	n/a	n/a
Huddinge	0.49	0.49	n/a	n/a
Linköping	0.60	0.56	0.02	0.63
Motala	0.48	0.45	0.18	0.42

*mSEAL* modified Skåne Emergency Department Assessment of Patient Load

*sICMED* short International Crowding Measure in Emergency Departments

*NEDOCS* National Emergency Department Overcrowding Score

* Ambulances waiting to offload patients not calculated

** Respirator/Trauma room variable not calculated

The AuROC was calculated based on 287 of 333 (86%) available time points for mSEAL and Occupancy rate and 160 of 202 (79%) time points for sICMED and NEDOCS with values between 0.82 and 0.97. Model results with suggested cutoffs and test characteristics that predict crowding are shown in Table 3.

**Table 3:** AuROC and optimal model cut-off to predict an assessed workload of 4.5

Model	AuROC (95% CI)	Cut-off*	Sensitivity	Specificity
**mSEAL**	0.91 (0.86–0.95)	4.8	0.87	0.78
**sICMED**	0.82 (0.50–0.99)	2	1.0	0.34
**NEDOCS**	0.97 (0.92–0.99)	90	0.93	0.93
**Occupancy rate**	0.90 (0.85–0.95)	1.2	0.87	0.82

*ED* Emergency Department

*mSEAL* modified Skåne Emergency Department Assessment of Patient Load

*sICMED* short International Crowding Measure in Emergency Departments

*NEDOCS* National Emergency Department Overcrowding Score

*AuROC* Area under the Receiver Operating Curve

* Based on Youden index

## Discussion

In this study, we derived and internally and externally validated the mSEAL score. Our findings indicated that mSEAL can be used to estimate ED crowding based on staff-perceived workload. The mSEAL score performed equal to the Occupancy rate and NEDOCS, and slightly better than sICMED based on their respective correlations and AuROC. Unfortunately, NEDOCS and sICMED could not be measured at two EDs due to limitations in the EHR data. With mSEAL we have the possibility to systematically measure crowding and evaluate efforts to reduce its effect on patient care. Additionally, the mSEAL model includes variables reported to the Swedish national ED registry and the National Board of Health and Welfare and has the potential of measuring crowding nationally, and also retrospectively [20, 21].

We derived the mSEAL score with the same process and data as the original SEAL model, but with the inclusion of only variables that could be retrieved at all hospitals. The mSEAL score thus includes two variables instead of four in the original SEAL score, and the additive value of mSEAL over a single measure such as Occupancy rate is probably small. However, the coefficient of determination scores were consistently better for mSEAL than for Occupancy rate at all hospitals. In situations where automatic measuring is not possible, Occupancy Rate may be a good alternative to mSEAL score at Swedish EDs.

Our results are similar to previous studies on both NEDOCS and ICMED. In a comparison of NEDOCS and the Emergency Department Work Index (EDWIN) at a single center in the US, Weiss et al. found an AuROC of 0.83 for NEDOCS [22]. When defining crowding as ambulance diversion, NEDOCS had an AuROC of 0.88 at four US hospitals [23]. The ICMED and NEDOCS models were validated against perceptions of crowding and danger at four UK hospitals with r-values of 0.73 (r^2^ = 0.53) and 0.77 (r^2^ = 0.59) respectively [10]. A study on sICMED at seven EDs in five countries showed moderate correlation with staff perception of crowding (r = 0.41, r^2^ = 0.17) and safety (r = 0.46, r^2^ = 0.21), with considerable variation between EDs [13].

There is no national consensus regarding when an ED is crowded in Sweden. To calculate the test characteristics of the mSEAL score we compared it against a dichotomized score of higher than 4.5 (crowded) or lower (non-crowded). The suggested model cutoffs in Table 3 is based on the Youden index which is the value with highest sum of sensitivity and specificity. Both cutoffs for NEDOCS (90) and OR (1.0) are close to the established values of 100 and 1.0 respectively, suggesting generalisability of an mSEAL score of 4.5 as an indication of crowding. Ideally, this cutoff should correspond to a level of crowding where patient care is compromised and morbidity and mortality increases and should be the focus of further research.

### Limitations

All model scores were calculated on data from EHRs that rely on manual input from staff of time-based metrics like *Time to physician*. There is thus a risk of input errors or delays that may be more pronounced during crowded periods.

Data was collected in the calendar months of September–November. Although some data suggest seasonal effects on ED boarding [24], others found no seasonal effects on ED visits [25, 26]. Since any seasonal effects would probably impact all scores similarly, we find it unlikely that they affected our results.

Staff caring for high acuity patients at the time point rated their workload as soon as possible afterwards, and if this was not possible within 1 h, the ratings were not recorded. This may decrease the sensitivity to crowding of the mSEAL model. We did not systematically track the number of unrated data due to high workload but it was a rare phenomenon in the three EDs where a researcher was present at a majority of the time points.

## Conclusions

The mSEAL model can measure crowding based workload in Swedish EDs with good discriminatory capacity and may be a tool to systematically evaluate crowding and help policymakers and researchers target its causes and effects. Occupancy Rate and NEDOCS are possible to measure in Swedish EDs and may be good alternatives to mSEAL.

## Data Availability

The datasets generated during and/or analysed during the current study are available from the corresponding author on reasonable request.

## Abbreviations

AuROC: Area under the receiver operating curve
ED: Emergency Department
EDWIN: Emergency Department Work Index
EHR: Electronic Health Record
ICMED: International Crowding Metric in Emergency Department
NEDOCS: National Emergency Department Overcrowding Score
MD: Medical Doctor
mSEAL: modified Swedish Emergency Department Assessment of Patient Load
SEAL: Swedish Emergency Department Assessment of Patient Load
sICMED: Simplified International Crowding Measure in Emergency Department
US: United States
