# Correction to: Validation of the modified Skåne emergency department assessment of patient load (mSEAL) model for emergency department crowding and comparison with international models; an observational study

**DOI:** 10.1186/s12873-021-00448-w

**Published:** 2021-04-16

**Authors:** Jens Wretborn, Håkan Starkenberg, Thoralph Ruge, Daniel B. Wilhelms, Ulf Ekelund

**Affiliations:** 1 Department of Emergency Medicine, Local Health Care Services in Central Östergötland, Linköping, Östergötland Sweden; 2 Department of Clinical Sciences Lund, Emergency Medicine, Faculty of Medicine, Lund University, Lund, Sweden; 3 Enköping Hospital, Region Uppsala, Sweden; 4 Department of Emergency Medicine Solna, Karolinska Institutet, Solna, Sweden; 5 Department of Emergency and Internal Medicine, Skåne University Hospital, Malmö, Sweden; 6 Department of Clinical Sciences Malmö, Faculty of Medicine, Lund University, Lund, Sweden; 7 Department of Biomedical and Clinical Sciences, Faculty of Health Sciences, Linköping University, Linköping, Sweden

**BMC Emerg Med 21, 21 (2021)**

**https://doi.org/10.1186/s12873-021-00414-6**

It was highlighted that in the original article [1] the Ethics approval and consent to participate section was incorrect. This Correction article shows the incorrect and correct Ethics approval and consent to participate section. The original article has been updated.

**Incorrect**

**Ethics approval and consent to participate.**

The study was approved by the regional ethics committé at Linköping University and Karolinska institutet.

**Correct**

**Ethics approval and consent to participate.**

The part of the study pertaining to the Linköping University Hospital and Motala Community Hospital was approved by the regional ethics committee at the county of Östergötland. The data from the Karolinska Hospital in Huddinge and Solna was part of a quality improvement initiative only including anonymised data, and as such is exempt from ethical approval in Sweden.
